# An Uncommon Case of Bowel Angioedema Due to Lisinopril

**DOI:** 10.7759/cureus.100319

**Published:** 2025-12-29

**Authors:** Cristina Vidal Carrion, Hannah Warshaw, Sree Uppalapati, Zubia Ahmed, Margi Desai

**Affiliations:** 1 Internal Medicine, Northeast Georgia Medical Center Gainesville, Gainesville, USA; 2 Internal Medicine, Philadelphia College of Osteopathic Medicine, Suwanee, USA

**Keywords:** ace inhibitor induced angioedema, ace inhibitor side effect, drug-induced angioedema, large bowel angioedema, small bowel angioedema

## Abstract

In general, angioedema can be caused by a hereditary C1 esterase deficiency or by an allergy-related cascade involving either a histamine or a bradykinin-induced cascade. Angioedema secondary to angiotensin-converting enzyme inhibitors (ACEi) typically induces an allergy-related angioedema and typically presents complications associated with the upper airway, and rarely presents intra-abdominally. This case discussed how a 54-year-old male with a recent diagnosis of hypertension presented with acute abdominal pain. He was started on a lisinopril-hydrochlorothiazide combination two days before the presentation. Differentials were considered for an acute abdomen, and a CT abdomen was obtained, which was significant for the thickening of the jejunum and sigmoid colon with surrounding mucosal edema and inflammatory changes. The patient was ultimately treated for bowel angioedema successfully with dexamethasone, diphenhydramine, and famotidine, and his lisinopril was discontinued. This case report aims to discuss and evaluate bowel angioedema in the setting of ACEi use and how it should be considered as a differential for an acute abdomen and its relevant empirical management with steroids due to the indistinguishable etiology of histamine or bradykinin mediated.

## Introduction

Angioedema (AE) is a self-limiting condition characterized by increased vascular permeability resulting in edema of the skin and mucosal tissues. It may arise from histamine-mediated allergic reactions or from bradykinin-mediated pathways, including hereditary C1 esterase inhibitor deficiency and angiotensin-converting enzyme inhibitor (ACEi)-related mechanisms [1]. Clinical manifestations include swelling of the face, lips, tongue, uvula, and upper airways, requiring emergency care as it can be life-threatening in severe cases [2,3]. Gastrointestinal involvement is uncommon but clinically significant, as it may mimic an acute abdomen and lead to unnecessary surgical intervention.

Histamine-mediated AE is the most common form and typically occurs within an hour of contact with allergen exposure. It usually presents with hives and can last for a few days. Bradykinin-mediated AE is more often associated with hereditary angioedema, C1 esterase inhibitor deficiency, or ACEi-related mechanisms. It usually does not have an association with hives upon presentation. AE secondary to ACEi typically induces a bradykinin-related AE and typically presents complications associated with the upper airway, with intra-abdominal manifestations occurring only rarely.

The pathophysiology of ACEi-induced angioedema (ACEi-AE) is fundamentally distinct from histamine-mediated AE. When ACE is inhibited, bradykinin degradation is impaired, leading to its accumulation along with other vasoactive peptides such as des-Arg9-bradykinin and substance P [4,5]. The interaction of these peptides with bradykinin receptors results in endothelial activation and leakage of plasma into the interstitial tissues, most notably in the submucosa and subcutaneous regions [4,5].

While superficial cases may not necessitate radiological evaluation, gut involvement requires careful consideration to avoid misdiagnoses, such as an acute abdomen, to prevent unnecessary surgical intervention. We present this case report to understand the imaging findings to help with a timely diagnosis and to review the pathophysiology that may have similar findings.

## Case presentation

A 54-year-old African American man with a history of asthma, diverticulitis, and cervical radiculopathy presented to the ED with abdominal pain. The patient has allergies to ciprofloxacin, piperacillin-tazobactam, and sulfamethoxazole-trimethoprim. He has an 8.6 pack-year smoking history. 

The patient was recently diagnosed with hypertension and started on a lisinopril-hydrochlorothiazide combination. The day before admission, after taking his medication, he experienced brief abdominal pain. The following morning, after his second dose, he developed severe, sharp, and dull abdominal pain, rated 10 out of 10, localized to the left abdomen, prompting his visit to the ED. He denied blood in his stool, diarrhea, constipation, fever, chills, chest pain, shortness of breath, hematuria, or dysuria. However, his wife noted a recent episode of hematuria during his last PCP visit, for which a renal ultrasound was planned. 

The differential diagnosis included diverticulitis, peptic ulcer disease, myocardial infarction, splenic infarction, pancreatitis, acute gastritis, enterocolitis, bowel obstruction, pyelonephritis, and nephrolithiasis. 

On examination, the patient was afebrile and bradycardic, with a normal respiratory rate, a blood pressure of 164/89 mmHg, and an oxygen saturation of 98% on room air. The physical exam revealed tenderness in the left abdomen with guarding. 

Laboratory investigations (CBC and CMP) were within normal limits, with a slightly elevated lipase, not consistent with pancreatitis. An EKG showed sinus bradycardia at 55 bpm, with a normal axis and intervals. Troponin levels were <3, ruling out NSTEMI. Urinalysis revealed 1 RBC, consistent with his prior hematuria episode. 

**Figure 1:**
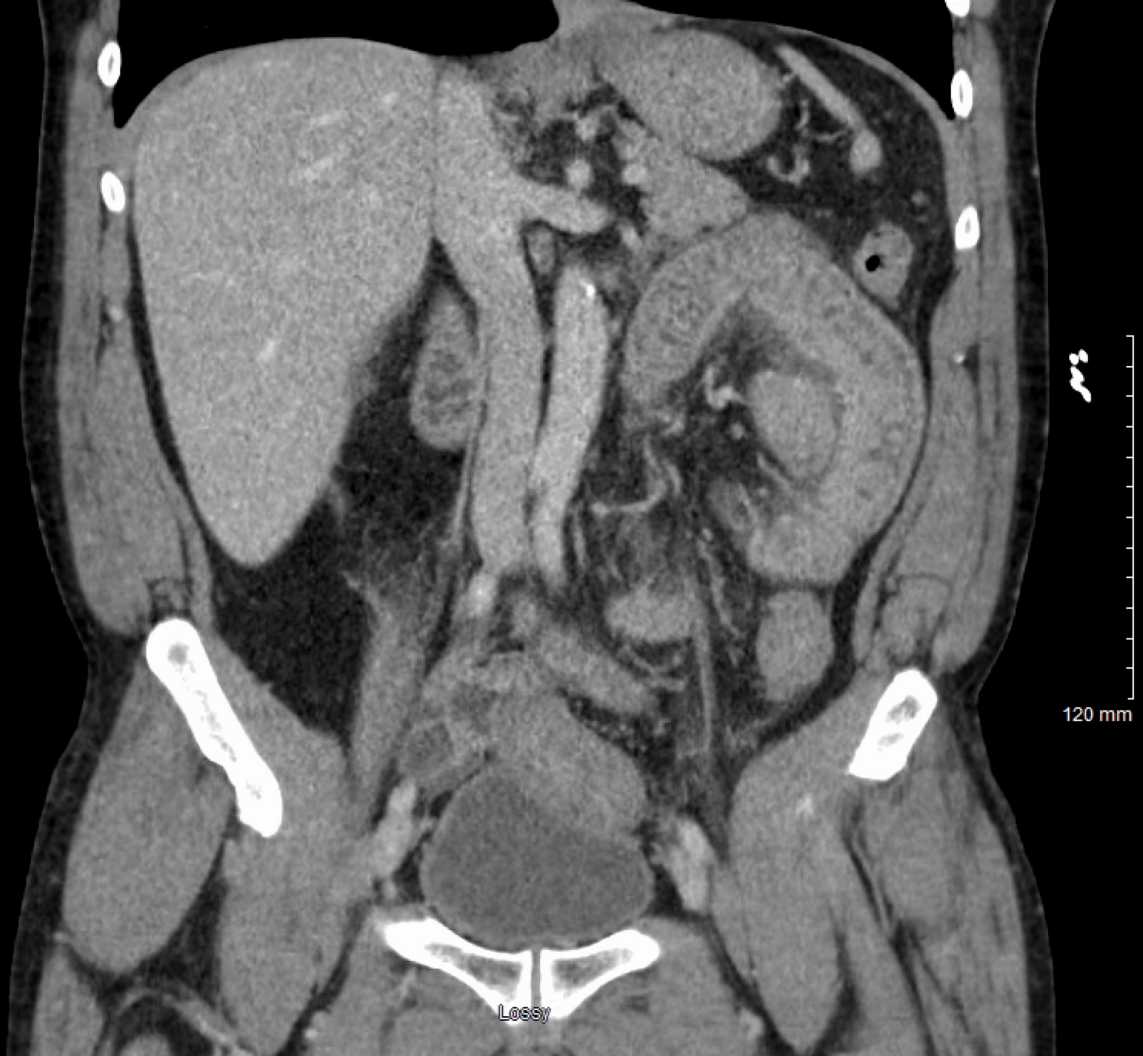
CT abdomen coronal view Demonstrated prominent thickening of the jejunum in the left upper quadrant with mucosal edema and surrounding inflammatory changes, as well as thickening of the sigmoid colon. These findings, in conjunction with adjacent free fluid (Figure 2) and the absence of obstruction, ischemia, or perforation, are characteristic of intestinal AE and help distinguish it from infectious enteritis, mesenteric ischemia, and acute diverticulitis.

**Figure 2:**
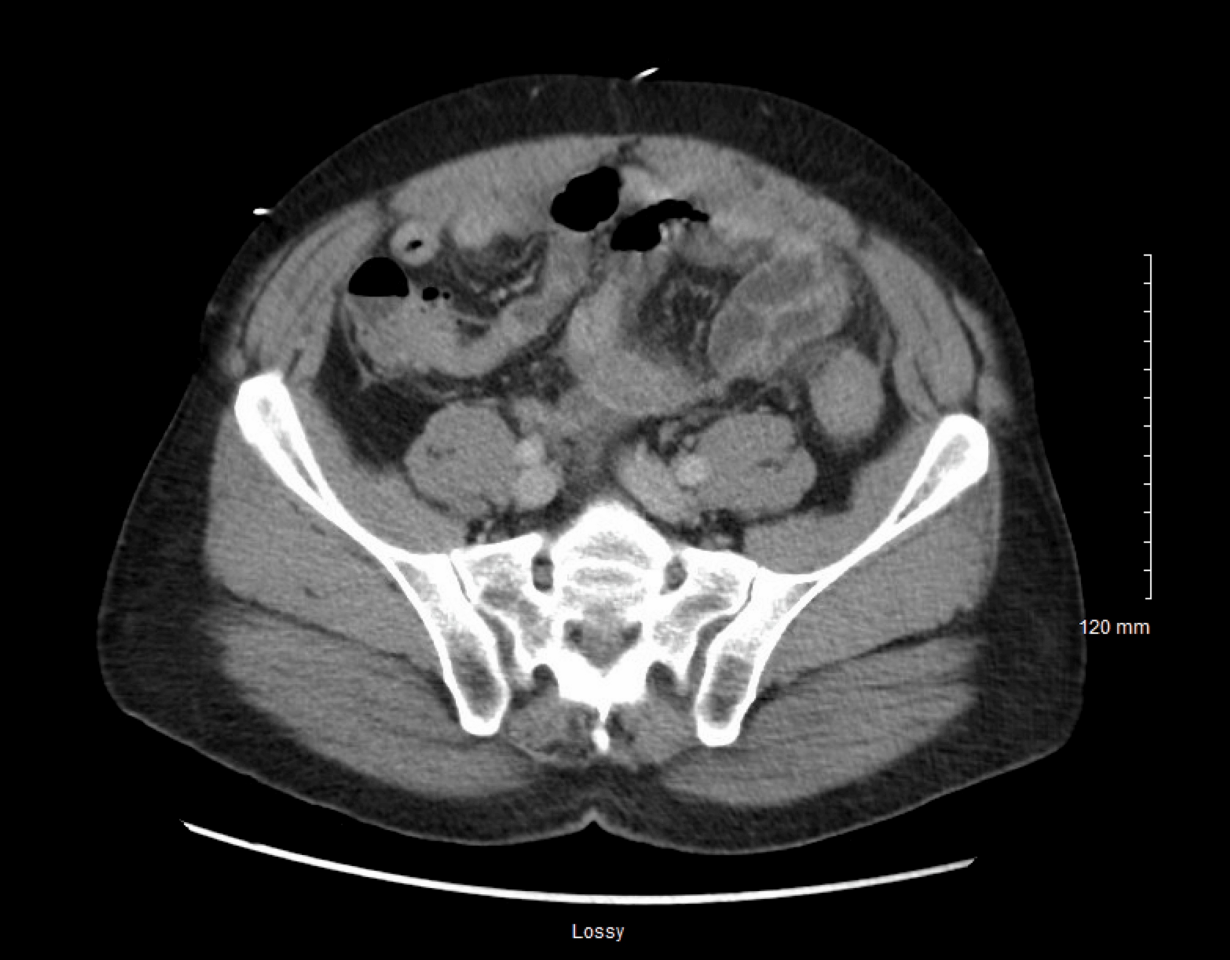
CT abdomen axial view This figure demonstrates moderate free intraperitoneal fluid within the pelvis and left paracolic gutter, without evidence of free air. The presence of free fluid in the absence of perforation supports the diagnosis of intestinal AE and helps distinguish it from hollow viscus perforation or inflammatory intra-abdominal processes.

A CT scan of the abdomen and pelvis revealed prominent thickening of the jejunum in the left upper quadrant with mucosal edema and surrounding inflammatory changes, as well as thickening of the sigmoid colon. There was moderate free fluid in the pelvis and left paracolic gutter, with no free air. The kidneys and ureters were unremarkable, with no hydronephrosis, stones, or solid masses. Colonic diverticula were noted without signs of acute diverticulitis (Figures 1, 2). 

Given the strong temporal association between symptom onset and initiation of lisinopril and the absence of a personal or family history suggestive of hereditary AE, the patient was diagnosed with intestinal AE involving the jejunum and sigmoid colon secondary to ACE inhibitor use. The CT findings were more consistent with AE. Bowel obstruction was ruled out, as there was no transition point, proximal bowel dilation, or fecalization of bowel contents. Similarly, the absence of vascular occlusion, pneumatosis intestinalis, or portal venous gas argues against mesenteric ischemia. Recognition of these distinguishing features is essential for non-specialists to differentiate bowel angioedema from more common causes of acute abdominal pain. 

Lisinopril was discontinued. The patient was treated empirically with dexamethasone, diphenhydramine, and famotidine. Atropine was administered for bradycardia. Complement levels, including C4 and C1 esterase inhibitor, were not pursued, given rapid clinical improvement following drug discontinuation. The patient’s symptoms improved, and he was discharged the following day.

## Discussion

ACEi-AE is a rare yet potentially life-threatening adverse effect of ACE inhibitors, with an estimated incidence ranging from 0.1% to 0.7% among patients receiving these medications [6]. Despite its relatively low frequency, ACEi-AE carries significant clinical implications due to its unpredictable nature and potential for rapid airway compromise. Between 1979 and 2010, AE was a contributing factor in 5,758 deaths in the United States, with Black patients accounting for a disproportionately high 55% of ACEi-associated fatalities [7,8]. While ACE inhibitors are widely used to treat hypertension, heart failure, and diabetic nephropathy, the risk of AE warrants heightened clinical awareness, especially given the increasing use of these drugs across diverse populations. 

AE has been found to affect those of African American background more than other ethnicities. While the mechanism is unknown, there have been a few reports that point to higher bradykinin sensitivity as the issue. One study has found lower urine levels of kallikrein in African American patients. As this substance is crucial in forming bradykinin, these patients are found to have lower levels of baseline bradykinin as well. This causes an upregulation of bradykinin receptors in the Black population. As an ACE inhibitor is introduced into the system, bradykinin degradation is inhibited, and sensitivity to the substance is increased due to the upregulation of its receptors compared to the non-African American population [9]. 

Importantly, clinicians should also recognize that ACEi-AE may present with atypical symptoms such as abdominal pain, nausea, and vomiting due to intestinal angioedema, which can mimic other conditions like mesenteric ischemia, small bowel obstruction, or bowel infarction [8]. This gastrointestinal manifestation, though less commonly reported, can lead to misdiagnosis and unnecessary interventions if not properly identified. 

This patient's risk factors prompted an evaluation for acute coronary syndrome. Although abdominal pain is an atypical presentation of NSTEMI, especially in men over 50, his normal troponins, ECG, and absence of chest pain made this diagnosis unlikely. 

The CT findings of jejunal wall thickening with mucosal edema, surrounding inflammatory changes, and moderate free pelvic fluid warranted a broad differential diagnosis. Potential considerations include infectious enteritis, ischemic bowel disease, inflammatory bowel disease, vasculitis, and even early neoplasms. Infectious etiologies usually present with fever, diarrhea, and systemic signs of infection, but none of these were observed in this patient. Bowel ischemia is more common in older patients with cardiovascular risk factors. The patient had a history of hypertension and presented with bradycardia, but typical features include pneumatosis intestinalis, portal venous gas, or mesenteric vascular occlusion on imaging. These hallmark findings were absent, making ischemic changes less likely. Diverticulitis was considered due to the presence of colonic diverticula, but was unlikely, as there were no pericolic fat stranding or signs of localized perforation. Vasculitis might present similarly, but is usually associated with systemic symptoms and elevated inflammatory markers, which were not present in this case.

Stepwise evaluation highlighted the temporal relationship between symptom onset and recent initiation of lisinopril. Imaging findings of segmental submucosal edema without obstruction, infection, or ischemia, combined with the absence of systemic inflammatory signs, supported the diagnosis of ACEi-AE. This approach demonstrates how careful integration of clinical history, laboratory evaluation, and imaging allows clinicians to systematically narrow the differential and reach an accurate diagnosis.

Therapeutic strategies for ACEi-AE are limited and primarily supportive. Due to the timeline and indistinguishable etiology of histamine- or bradykinin-mediated angioedema, this patient was treated for both. ACEi-AE does not respond to conventional anti-allergic treatments such as antihistamines, corticosteroids, or epinephrine, which are ineffective in non-histaminergic angioedema [10].

The key element of management is immediate discontinuation of the offending ACE inhibitor, as continued use significantly increases the risk of recurrent and potentially more severe episodes [9]. Although therapies effective in hereditary angioedema, such as C1-esterase inhibitor (C1INH) therapy, have been trialed in ACEi-AE, their efficacy has been inconsistent. In some clinical settings, C1INH therapy failed to outperform placebo in terms of reducing time to symptom resolution, particularly when used alongside concomitant use of antihistamines and steroids [10]. Tranexamic acid (TXA), a low-cost alternative that has been studied for its action against plasmin activation, causing a downstream inhibition of coagulation factors, like Factor XII, may reduce the production of bradykinin and ultimately reduce symptoms of AE [11]. In the same report, it was shown that TXA was associated with a decreased need for intubation and reduced length of hospital stay. It is also important to note that most therapeutic interventions have been evaluated primarily in patients with airway compromise. Due to the rarity of gastrointestinal involvement, most treatments and supportive measures have only been studied in that context. Clinical improvement in ACEi-AE is most reliably attributed to discontinuation of the ACE inhibitor rather than adjunctive therapies.

The diagnosis of ACEi-AE is often clinical, supported by a history of ACE inhibitor use and the absence of response to anti-allergic medications. ACEi-AE is bradykinin-mediated, making immediate discontinuation of lisinopril the central therapeutic step, while corticosteroids and antihistamines are used empirically and are not reliably effective. Delays in diagnosis can increase morbidity, especially when alternative causes are erroneously pursued. Increased awareness among clinicians is critical, particularly in emergency and primary care settings, where initial presentations often occur. As there are only a handful of cases discussing intestinal angioedema, recognizing the ethnic predispositions, especially among Black and Hispanic populations, may also aid in early identification, prompt management, and appropriate risk counseling. 

## Conclusions

ACEi-AE presents a unique diagnostic challenge due to its variable presentation and bradykinin-mediated pathophysiology. Although rare, delayed recognition may result in life-threatening complications. Diagnostic imaging plays a crucial role in identifying atypical manifestations, particularly gastrointestinal involvement that may mimic an acute abdomen. Timely diagnosis, immediate drug cessation, and increased clinical awareness remain the most effective strategies for management. In patients presenting with unexplained acute abdominal pain, careful review of medication history is essential to ensure timely diagnosis and to avoid unnecessary invasive interventions. Further research is needed to refine therapeutic strategies and address the disparities observed among high-risk populations.
